# Conservative Treatment of Chronic Achilles Tendinopathy: A Systematic Review

**DOI:** 10.3390/jfmk4030046

**Published:** 2019-07-22

**Authors:** Vito Pavone, Andrea Vescio, Giuseppe Mobilia, Sara Dimartino, Giovanni Di Stefano, Annalisa Culmone, Gianluca Testa

**Affiliations:** Department of General Surgery and Medical Surgical Specialties, Section of Orthopedics and Traumatology, A.O.U. Policlinico-Vittorio Emanuele, University of Catania, Via Santa Sofia 78, 95123 Catania, Italy

**Keywords:** Achilles tendinopathy, Achilles tendon, conservative treatment, ESWT, Extracorporeal shockwave therapy, platelet rich plasma, PRP, leukocyte- and platelet-rich plasma, L-PRP, pharmacology therapy

## Abstract

Achilles tendinopathy is a common musculoskeletal disorder. Athletes, runners and jumpers, and the sedentary are frequently affected. Numerous are the therapeutic choices to manage these kinds of disorders. The aim of this review is to analyze the available literature to document the up-to-date evidence on conservative management of Achilles tendinopathy. A systematic review of two medical electronic databases was performed by three independent authors, using the following inclusion criteria: conservative treatment consisted of pharmacologic, physical therapy without operative treatment, with more of 6 months symptoms and a minimum average of 6-months follow-up. Studies of any level of evidence, reporting clinical results, and dealing with Achilles tendinopathy and conservative treatment were searched for. A total of *n* = 1228 articles were found. At the end of the first screening, following the previously described selection criteria, we selected *n* = 94 articles eligible for full-text reading. Ultimately, after full-text reading and a reference list check, we selected *n* = 29 articles. Achilles tendinopathy is a frequent musculoskeletal disorder and several conservative treatments have been proposed, but no therapy is universally accepted, except for eccentric exercise training, which is the gold standard and a commonly used protocol.

## 1. Introduction

Achilles tendinopathy is a common musculoskeletal disorder [1]. This pathology affects athletes, runners, and jumpers, and some patients between 30 and 55 years of age who do not participate in sports [2]. The annual incidence of tendinopathy is up to 9% among runners and up to 30% in people who are not physically active [3,4]. Some authors have considered the vascular anatomy theory, i.e., that in susceptible individuals, the insufficient blood support by an arterial branch from the peroneal artery could be insufficient to prevent degeneration and facilitate healing, and at the same time, have also considered choke zones, defined as the area between two vascular territories with narrow anastomotic vessels, which may reduce the tendon capacity for self-repair [5], representing a greater risk of rupture [5]. There are many risk factors and they can be either intrinsic or extrinsic risk factors. Intrinsic risk factors include sex, advanced age, and body mass index [2,6,7]. They could be correlated with hereditary factors or a genetic predisposition and endocrine, metabolic, and rheumatological diseases such as diabetes mellitus, hemochromatosis, amyloidosis, or rheumatoid arthritis [6,7,8,9]. Extrinsic risk factors include the factors that could alter the course and the prognosis of the disease, for example drugs such as glucocorticoids, statins, and some antibiotics such as fluoroquinolones and aromatase inhibitors [5,6,7]. Based on this assumption, functional overload could lead to a cumulative force on the tendon [10,11,12], while pronation and excessive supination can determine traction, because of incorrect neurological adaptations in the tendon and their further adjustments [10,11]. All of these factors should be considered at the appearance of pain or in the subclinical presentation to reach the correct diagnosis [7]. According to Mafulli et al. [6], tendinopathy is defined by the following triad: pain, swelling at the tendon, and functional impotence. In the initial phase, pain appears during and at the end of a sports activity and then it becomes permanent in the daily routine [12]. For tendinopathy, different conservative treatments are taken into account, but because they are heterogenous, it is difficult to identify a gold standard. Surgery is considered when the pathology persists for at least 6 months [13]. The aim of this systematic review is to analyze the last 10 years of available literature to document the up-to-date evidence on conservative treatment of chronic Achilles tendinopathy.

## 2. Materials and Methods

### 2.1. Study Selection

PubMed and Science Direct databases were systematically reviewed by three independent authors (VA, MG, and DS) from their date of inception to 14 April 2019, according to the guidelines of the Preferred Reporting Items for Systematic Reviews and Meta-Analyses (PRISMA) [14]. The following search was entered: “(Achilles Tendon disease OR chronic Achilles Tendinopathy OR Achilles Tendinosis) NOT (Achilles Tendon rupture OR Achilles Tendon lesion) AND (conservative treatment OR pharmacologic treatment OR pharmacology OR physical therapy) NOT (surgical treatment OR surgery OR suture)”. The search was for a timeframe within the last 10 years (2008–2019). From each included original article, a standard data entry form was utilized to extract the number of patients, mean age at treatment, sex, type of treatment, minimum time of symptoms, duration of treatment, complication rate, and complication type, as well as the follow-up and year of the study. The quality assessment of the studies was performed in duplicate by two independent reviewers (VA and MG). Conflicts about the data were resolved by consultation with a senior surgeon (PV).

### 2.2. Inclusion and Exclusion Criteria

Eligible studies for the present systematic review included conservative Achilles tendinopathy treatment in last 10 years. The initial titles and abstract screening was performed using the following inclusion criteria: conservative treatment consisting of pharmacologic and physical therapy without operative treatment, symptoms with more than 6 months duration, and a minimum average follow-up of 6 months. The exclusion criteria included patients with primary or secondary surgical treatment, symptom duration of less than 6 months, and animal trials. We also excluded all the remaining duplicates, as well as articles dealing with other topics, articles not in the English language, and those without an accessible abstract. Reference lists were also hand-searched for further relevant studies. Abstracts, case reports, conference presentations, editorials, and expert opinions were also excluded. 

## 3. Results

### 3.1. Included Studies

**Figure 1:**
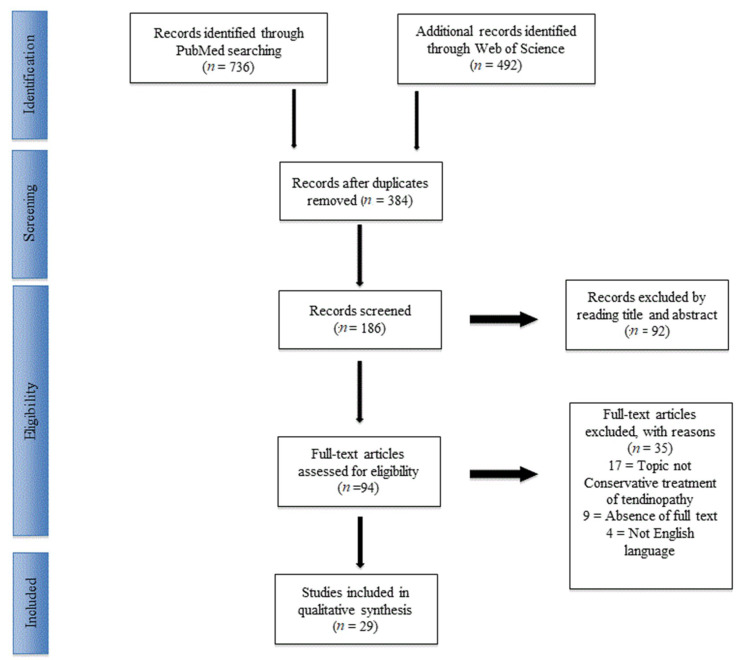
PRISMA (Preferred Reporting Items for Systematic Reviews and Meta-Analysis) flowchart of the systematic literature review.

There were 1228 articles that were identified. After excluding duplicates, 384 articles were selected. After the first screening and following the previously described selection criteria, 94 articles were eligible for full-text reading. Meta-analyses or systematic reviews were excluded. Ultimately, after full-text reading and checking the reference list, 29 articles were selected based on previously written criteria. These 29 studies comprised randomized controlled human trials (RCTs) and prospective and retrospective cohorts or series studies. A PRISMA [14] flowchart of the selection method was provided at the screening (Figure 1). The included articles [15,16,17,18,19,20,21,22,23,24,25,26,27,28,29,30,31,32,33,34,35,36,37,38,39,40,41,42,43] mainly focused on physical exercise, platelet rich plasma (PRP), and leukocyte- and platelet-rich plasma (L-PRP), extracorporeal shockwave therapy (ESWT), topical and systemic pharmacology therapy, or combination therapies. The main findings of the included articles are summarized in Table 1.

### 3.2. Physical Exercise

All the included studied reported improvements in functional outcome scores and in patient satisfaction, and a decrease in pain after eccentric loading exercises (ECC) treatment [15,16,17,18,19,20,21,22,23,24,25,26,27,28,29] (Table 1) compared to other training protocols [15,16] and intense pulsed light (IPL) [17], which were found to be similar. Hostmann et al. [18] recorded major pain improvements with vibration training compared to ECC, which was similar results reported by Zhang et al. [19], who documented a better VISA-A score in the acupuncture group compared to the ECC group. Five articles [20,21,22,23,24] compared the ECC protocol to the combination of ECC and adjuvant treatment. Patient outcomes and symptomatology were improved when ECC was associated with prolotherapy, [20] photobiomodulation, [21] oral supplementation, [22] and soft tissue treatment (Astym) [23]. De Jorge et al. [24] did not find any improvement in outcomes using night splints in addition to ECC. 

### 3.3. Platelet Rich Plasma and Leukocyte- and Platelet-Rich Plasma

Deans et al. [30] showed an increased in the quality of life and functional outcomes in patients treated with PRP, but de Vos [31] in 2010 and de Jong et al. [32] in 2011 reported no significant difference in improvement using the functional scores at 6, 12, and 24 weeks and at 1 year when PRP was compared to the ECC and placebo groups. Among patients who were treated with L-PRP, 91.6–93.0% [33,34] were satisfied after treatment and 89% returned to sport [33] with no Achilles tendon rupture [34] (Table 1).

### 3.4. Extracorporeal Shockwave Therapy

Six articles [35,36,37,38,39,40] explored the use of extracorporeal shockwave therapy (ESWT) to treat Achilles insertional and non-insertional tendinopathy, and all reported successful outcomes. There was no major advantage with the addition of an ultrasound for guiding shock waves [35]. At the 12-month follow-up, Pavone et al. [36] reported that 65.0% of patients did not complain about pain, 27.5% of patients returned to normal daily activities and sports despite residual pain, and three patients still had pain (pain score (Visual Analog Scale) > 4). Rompe et al. [37] recorded that 64% of subjects treated with ESWT achieved a level of 1 (completely recovered) or 2 (much improved) on the Likert scale after treatment (Table 1).

### 3.5. Topical and Systemic Pharmacologic Therapy

Pharmacological therapy was investigated in three of the included articles. Wetke et al. [41] described the results of topical glucocorticoids (GCS) in a cohort of 113 subjects and reported good short-term effects and a similar outcome in a long-term training group. Lynen et al. [42] compared two peritendinous hyaluronic acid (HA) injection protocols to ESWT and highlighted greater treatment success in Achilles midportion tendinopathy in pain at 4 weeks (*p* = 0.0304), 3 months (*p* = 0.0030), and 6 months (*p* = 0.0018) with the HA protocol. Maquirriain et al. [43] recommended etoricoxib as opposed to diclofenac because significantly fewer side effects were reported (0% and 14.2%, respectively, *p* = 0.037; Table 1).

## 4. Discussion

Conservative treatments for Achilles tendinopathy to date are exercise with eccentric loading, pharmacological therapies, pressure manipulation (Astym therapy), injections therapies with GCS, HA, and PRP, and ESWT.

Training with eccentric loading exercises is a treatment for the middle part of Achilles tendinopathy and it is considered to be the gold standard [6]. ECCs have been proposed to promote collagen fiber cross-link formation within the tendon by facilitating tendon remodelling [43]. Henriksen et al. [44] observed the changes in force oscillations and electromyographic amplitudes, which have distinctive biomechanical features in eccentric loading exercises, and they play a role in stimulating fibroblasts. However, the eccentric exercises could affect nervous system sensitization and enhance healthy tissue tolerance, rather than cause degeneration or dysfunctional tissue [45]. Yu et al. [46] confirmed that eccentric is more effective than concentric strengthening for reducing pain and improving function in patients with Achilles tendinopathy. In a study by Stasinopoulos [16], an exercise program based on the Alfredson protocol was superior to the Stanish model, and Vanderplus et al. [29] showed good results in a long-term follow-up study. While the success of these treatments is well-known, these studies are heterogeneous because, even if the Alfredson protocol could be standardized, it is commonly undermined by the increasingly selective exercises. For example, Verrell et al. [25] modified the traditional Alfredson protocol to incorporate body weight loading for at least 15 to 20 s and a 6-week dosage period, and they achieved a substantial decrease in pain and a high degree of effectiveness. Some authors found better outcomes by following different protocols. Rikke Beyer et al. [15], for example, used heavy slow resistance training (HSR) and showed good results and a greater degree of patient satisfaction, but they did not include a long-term follow-up. Thus, further trials that include long-term follow-ups are needed. Research suggests that if eccentric loading exercise is combined with other non-invasive treatment modalities, there are significant improvements in outcome measures. The injection of a solution of hypertonic glucose, called “prolotherapy”, promotes pro-inflammation reactions followed by collagen deposition, and stimulates neovascularisation in tendinosis [20]. In association with ECC, this could be one of most prospective adjuvant therapies, however, a small increase in cost and temporary patient discomfort was described [20]. Conversely, many studies such as Jong et al. [24] showed that after a 1-year follow-up, there was no difference in functional outcomes between treatment with eccentric loading exercises or a combination of eccentric loading exercises and the use of a night splint. Recently, some authors have tried to improve the effectiveness of eccentric loading exercises by combining them with some orthotic devices [27], but the effects are not better than for patients who followed only the physiotherapy treatment.

De Vos [31] investigated the clinical use of a PRP injection and found that there was no benefit in pain and function. De Jorge [32] also showed no clinical or sonographic benefit when compared with secondary outcomes, and this treatment has limits related to its composition. However, Guelfi et al. [34] showed beneficial effects and a low complication rate after a single L-PRP injection to heal mid-portion chronic recalcitrant Achilles tendinopathy (CRAT) in a large cohort of patients with mid- to long-term follow-ups, without eccentric loading exercises training.

GCS injections are another adjunct that are used in clinics. These injections around the Achilles tendon are controversial because the exact location of the inflammation is rarely found [47]. Moreover, in some cases it is not recommended because of the risk of tendon rupture [48]. To date, GCS infiltration is also not recommended, but studies such as that by Wetke [41] suggest using it in association with eccentric loading exercises in patients who are unable to begin or continue training because of intolerable pain. HA injection is another controversial treatment [42]. Pain was observed to change significantly at 3 months post-treatment. Because this study was the first to analyze the results after an HA injection, the reliability of these results are unknown. Another treatment is prolotherapy, a simpler injection that does not require an ultrasound. A solution of hypertonic glucose and local anesthetic is injected alongside the painful areas of the tendon [12] to stimulate inflammation, which is followed by collagen deposition [49]. A study of glucose-lignocaine injections versus saline injections into injured rat knee ligaments has shown additional thickening of the healing ligaments, but no differences in biomechanical properties [50]. Yellan [20] compared the effectiveness of eccentric loading exercises with prolotherapy injections and found that the mean functional outcome score improvements are greater with the combined treatment compared to only eccentric exercise. However, the proportion of patients who achieve important improvements in the functional outcome scores was similar for all groups. In this case, a more in-depth clinical trial is needed to evaluate these findings.

The mechanical stimulus provided by ESWT can aid in initiating tendon regeneration in tendinopathy by promoting pro-inflammation. This was confirmed by a microdialysis study that quantified the concentration of interleukins and metalloproteases after treatment with ESWT [51,52]. In this study, Waugh et al. [52] analyzed the in vivo the biological response to extracorporeal shockwaves, and they showed that in the dialysated tendon, there were some predominant cytokines, some of which had an elevated concentration immediately after ESWT, and then remained significantly elevated for several hours post-ESWT. The efficacy of ESWT is strictly related to energy level, the number of impulses, and the range of action used during treatment, and these indications must be taken into consideration. ESWT outcomes were better in patients without Haglund’s deformity compared to patients with Haglund’s deformity [53]. However, different studies showed that there were no major advantages to be had by adding an ultrasound to guide shock waves [35]. Pavone [36] and Saxena [39] suggested that using a low-energy radial shockwave is more convenient. It has been demonstrated that ESWT treatment produces better results than the wait-and-see policy, and when it is combined with eccentric training [54], ESWT treatment does not preclude surgery and may even improve the tendon’s condition for surgery [38].

Another approach to use in Achilles tendinopathy is IPL. The first experimental studies showed that in vitro, there were potential physiological effects in the treatment of patients with Achilles tendinosis. However, Hutchinson [17] conducted a double-blind randomized controlled trial (RCT) and showed that there was no significantly different outcomes in the control group, and that IPL was not effective for acute treatment. In addition, laser-induced photobiomodulation (PBM) was shown to induce changes in membrane permeability, stimulate cytokine reactions, release growth factors, and upregulate ATP, nitric oxide (NO), and oxidation-reduction (REDOX) signaling, and thereby increase metabolism and cell proliferation [55,56]. According to Tumilty et al. [21], this technique showed statistically significant improvements. Moreover, this study suggests that PBM (using the right parameters as an adjunct to minor sessions of eccentric exercise) can provide an added benefit at 12 weeks. In addition to ECC treatment, orally administered drugs provided different results, depending on the drugs that were used. Praet et al. [22] proposed an RCT to treat tendinopathy with magnesium and vitamin E, because the author self-administered the drug and the symptoms resolved. Specific collagen peptides with high glycine content may accelerate the clinical benefits of a well-structured calf-strengthening and return-to-running program in patients with unilateral or bilateral chronic Achilles tendinopathy symptoms [22]. 

Another approach is Astym therapy, which is a noninvasive therapeutic approach that is used for soft-tissue dysfunction. It works via handheld instrumentation and it is applied topically to locate the underlying dysfunctional soft tissue and then transfer a certain amount of pressure to the dysfunctional tissue using specific protocols and patterns that were developed based on scientific and clinical study [56,57]. Mccormack [23] showed significant improvements in pain over the short- and long-term, but to evaluate the real benefits of this therapy on a variety of musculoskeletal disorders, more studies are needed. 

According to the data available in the literature, the evidence does not support any approach strongly. The eccentric loading exercises are commonly investigated and considered the benchmark of Achilles tendinopathy conservative treatments and the use of adjuvant therapies such as prolotherapy, photobiomodulation, oral supplementation, and soft tissue treatment seems to improve the fuctional outcome of the patients, but additional studies are needed. We encourage high-profile clinical studies to examine more recent techniques.

## 5. Conclusions

Achilles tendinopathy is a common musculoskeletal disorder and several conservative treatments have been proposed, but no therapy is universally accepted, except for eccentric exercise training, which is the gold standard and a commonly used protocol.

## Figures and Tables

**Table 1:** The main findings of the studies. ECC= eccentric loading exercise; COE= concentric loading exercise; PRP= platelet rich plasma; L-PRP= leukocyte- and platelet-rich plasma; ESWT= extracorporeal shockwave therapy; ultrasonography= US; GCS= glucocorticoids; HA= hyaluronic acid.

Ref	Authors (Year of Publication)	No. Of Patients	Treatment Groups	Results Summery
[15]	Beyer et al. (2015)	58	ECC vs. heavy slow resistance training (HSR)	Both groups had improvement in the short- and long-term ranges over the baseline (*p* < 0.05), but no differences between the treatments were recorded.
[16]	Stasinopoulos et al. (2013)	41	Alfredson ECC vs. Stanich ECC	Alfredson protocol was superior to Stanish model in reducing pain and improving function outcomes. Both groups had improvements at the 6-month follow-up over the baseline.
[17]	Hutchison et al. (2013)	47	Intense pulsed light vs. ECC	No differences between the groups at 1 year follow-up.
[18]	Hostmman et al. (2013)	58	Vibration traning vs. ECC vs. control	Pain reduction in the vibration training and ECC groups compared to the control. In the musculotendinous junction, ECC cohort had a 66.6% reduction in pain. The vibration-training group did not experience any change in pain at the musculotendinous junction, therefore, vibration training 0%, while the control group had an increase of the 73.3%.
[19]	Zhang et al. (2012)	64	Acupuncture vs. ECC	At the 8-week follow-up, there was superior improvement of the pain score acupuncture group compared to ECC (67.1 points and 48.5, respectively).
[20]	Yelland et al.(2009)	43	Proloteraphy + ECC vs. ECC	At the short- and long-term follow-up, there was a superior increase amongproloteraphy + ECC compared to the others. At the 1 year follow-up, the proloteraphy + ECC group had an improvement of 86% for the functional score, with 73% for ECC. In combined treatment there was early recovery.
[21]	Tumilty et al. (2015)	80	Photobiomodulation +ECC vs. ECC	Photobiomodulation + ECC group showed statistically significant improvements over ECC only in functional score.
[22]	Praet et al (2019)	18	ECC + Oral supplementation (OS)	In the OS group, there was an early return to sport and improvements in functional outcome.
[23]	McCormack et al. (2016)	16	ECC vs. ECC + Astym Treatment	After 12-, 26- and 52-week follow-ups, Astym + ECC patients showed better outcomes than ECC groups for insertional tendinopathy. Both groups recorded significant improvements in pain over the baseline, but there was no difference between cohorts, except at the 12-week intervention period in the combined group.
[24]	de Jorge et al. (2010)	58	ECC vs. ECC + Night splint	There was an improvement in functional score in at the 3-month and 1-year follow-ups over the baseline in the ECC group and the ECC + night splint group. There was no significant difference found in increases in pain score.
[25]	Verral et al. (2011)	190	ECC	There was a reduction of pain after 12 weeks, in 6 to 14 months of treatment (*p* < 0.01 compared to the baseline).
[26]	Ram et al. (2013)	45	ECC vs. Control	There was superior patient satisfaction for the ECC group compared to the control. No statistically significant assessments at 12 weeks in groups of the satisfied and not satisfied patients were made, nor were improvements recorded at the subsequent follow-up. There was better color Doppler activity during their second ultrasound.
[27]	Yu et al, (2013)	32	ECC vs. COE	Both groups had improvements over the baseline (*p* < 0.05)
[28]	Stevens et al. (2014)	28	ECC vs. “Do-as-tolerated” protocol	Both groups presented clinically and statistically significant improvements at the 6-week follow-up over the baseline.
[29]	Van der Plas et al. (2011)	46	ECC	There was improvement of pain and functional scores after 1 year of treatment (*p* < 0.001), and at the 5-year follow-up (*p* < 0.01). 39.7% of the patients were completely pain-free at the follow-up and 48.3% had received one or more alternative treatments.
[30]	Deans et al. (2012)	26	PRP	There was improve quality of life and functional outcomes. 5 had worse symptoms, there was 1 rupture, and 2 found it difficult to work.
[31]	de Vos et al. (2010)	54	PRP vs. ECC+Placebo	At the 6-, 12-, and 24-week follow-ups, there was no significant difference in the improvement of functional outcomes between the 2 treatment groups.
[32]	de Jonge et al (2011)	54	PRP+ECC vs. ECC+Placebo	At the 6-month and 1-year follow-ups, there was not a significant difference in clinical or sonographic assessments after a PRP injection. In both groups, neovascularization continued decreasing after the increase at the 12-week follow-up.
[33]	Filardo et al. (2014)	27	L-PRP	89% returned to sport and 93% were satisfied patients. In the unsatisfied patients, 1 patient had a corticosteroid injection and 2 patients had surgical intervention.
[34]	Guelfi et al. (2014)	83	L-PRP	At the final follow-up there was an improvement of functional outcome (*p* < 00.1 respect baseline). 91.6% were satisfied after 1 injection, the remaining 8.4% had a second PRP injection. No reported Achilles tendon ruptures.
[35]	Njawaya et al (2017)	27	US+ESWT vs. ESWT	Similar results were recorded in the 2 groups with, no major advantage seen in the addition of an ultrasound for guiding shock waves.
[36]	Pavone et al. (2016)	40	ESWT+ ECC vs. ECC	At the 12-month follow-up, 65.0% of patients did not complain about pain, 27.5% of patients got back to normal daily activities and sports despite residual pain, and 3 patients still complained about pain (VAS > 4). Statistically significant differences in the pain score and in the functional outcome results were observed as well as in the functional outcome.
[37]	Rompe et al. (2008)	50	ESWT vs. ECC	At the 4-month follow-up, no significant difference was seen in the functional assessments between the two groups. ESWT patients showed better outcomes than ECC subjects (*p* < 0.020)
[38]	Vulpiani et al. (2009)	105	ESWT	60 days after the end of the treatment, there was a significant improvement over the baseline in pain score.
[39]	Saxena (2011)	60	ESWT	At least 1 year after treatment, there were significant improvements (78.38%) of satisfied patients treated with the low-energy radial shockwave devices.
[40]	Taylor et al. (2016)	56	ESWT	In both non- and insertional tendinopathies, there were improvements in the mean pain scores at rest and on activity.
[41]	Wetke et al. (2014)	113	GCS vs. training	There were good short-term effects, but no significant long-term effects. 26% of patients had training only, 58% had one supplementary injection, 14% had two injections, and 2% had three injections. 2 subjects had flare-ups more than 24 h after GCS injection
[42]	Lynen et al. (2016)	59	HA vs. ESWT	90 days after the treatment, the HA group had greater treatment satisfaction than the standard ESWT in terms of pain (*p* = 0.0030). Similar findings for HA were also observed at 4 weeks (*p* = 0.0304) and 6 months (*p* = 0.0018).
[43]	Maquirriain et al. (2013)	56	Eterocoxib vs. Diclofenac	Over the 7-day treatment period, both groups had improvements over the baseline (*p* < 0.001). The analgesic effect averaged etoricoxib= 56.4% and diclofenac 50.6% (*p* = 0.64). The etericoxib groups recorded less side effects than those in the diclofenac group (0% and 14.2%, respectively, *p* = 0.037)
